# AVAiLABLE NIS – AVASTIN® in lung cancer treatment in routine oncology practice in Germany

**DOI:** 10.1186/s12885-019-5618-0

**Published:** 2019-05-10

**Authors:** Mark-Oliver Zahn, Dominik Linck, Christoph Losem, Christian Gessner, Holger Metze, Vincent E. Gaillard, Hans Werner Tessen

**Affiliations:** 1 Onkologische Schwerpunktpraxis Goslar, Kösliner Str. 14, 38642 Goslar, Germany; 2 Onkologische Schwerpunktpraxis Euskirchen, Gottfried-Disse-Str. 42, 53879 Euskirchen, Germany; 3 MVZ für Onkologie und Hämatologie im Rhein-Kreis Neuss, Am Hasenberg 44, 41462 Neuss, Germany; 4 Pneumologische Schwerpunktpraxis mit pneumologischer Onkologie Leipzig, Tauchaer Str. 12, 04357 Leipzig, Germany; 5 Pneumologie des Rhön-Klinikums Frankfurt/Oder, Müllroser Chaussee 7, 15236 Frankfurt/Oder, Germany; 6 F. Hoffmann-La Roche Ltd, Product Development Medical Affairs, Grenzacherstrasse, 4070 Basel, Switzerland

**Keywords:** Advanced non-small-cell lung cancer, Adenocarcinoma, Bevacizumab (Avastin®) plus chemotherapy, Post-authorization study

## Abstract

**Background:**

Bevacizumab (Avastin®), a recombinant humanized monoclonal antibody, in combination with platinum-doublet chemotherapy has become a routine treatment for advanced non-small-cell lung cancer (NSCLC). The post-authorization, non-interventional study ‘AVAiLABLE’ assessed the effectiveness and safety of bevacizumab combined with chemotherapy as first-line treatment.

**Methods:**

Nine hundred and eighty-seven adult patients (mean age 61.5 years, 59.8% male) with non-resectable advanced, metastatic or recurrent, predominantly non-squamous NSCLC were evaluated at 185 sites across Germany. 72.8% of the patients had stage IV disease at start of observation, 90.1% had histologically confirmed adenocarcinoma and 80.8% met the bevacizumab label ‘NSCLC other than predominantly squamous cell histology’. According to bevacizumab label, chemotherapy plus bevacizumab was recommended, followed by bevacizumab maintenance therapy. Effectiveness endpoints included response rates and progression-free survival (PFS); safety endpoints comprised adverse drug reactions (ADRs). Patients were followed until progression or intolerable toxicity. Data were evaluated by descriptive statistical methods.

**Results:**

Median PFS was 7.4 months (95% CI: 7.1; 8.4), overall response rate (ORR) 45.6% and disease control rate (DCR) 75%. The majority of patients (72.7%) achieved partial response or stable disease. Complete response was reached by 2.3%. 33.6% of patients experienced an ADR of grade ≥ 3. Bevacizumab-related ADRs of grade ≥ 3 occurred in 5.7% of patients, with the highest incidence for leukopenia, neutropenia, and hypertension.

**Conclusions:**

Results of the non-interventional study ‘AVAiLABLE’ confirmed the effectiveness and safety of bevacizumab in combination with platinum-based chemotherapy as first-line treatment for advanced NSCLC in accordance with previous studies. No new safety signals were identified. Maintenance therapy with bevacizumab was well tolerated and safe even over extended periods (> 20 cycles).

**Trial registration:**

ClinicalTrials.gov Identifier: NCT02596958; registered on 4 November 2015.

## Background

Lung cancer is the leading cause of cancer-related deaths worldwide. In Germany, the prognostic incidence of lung cancer for the year 2020 is 36,200 for men and 23,700 for women [1]. In the United States, the estimates for 2018 are a total of 234,030 new cases and 154,050 deaths [2]. The most common type of pulmonary malignancies is non-small-cell lung cancer (NSCLC) with more than 80% of all cases. Despite numerous research activities and therapeutic efforts, the prognosis is still unfavorable due to late stage diagnoses with distant metastases in about 70% of patients. The 5-year survival rate varies markedly depending on the stage at diagnosis, decreasing from 60.1% to 33.4% to 5.5% for patients with local, regional, and distant stage disease, respectively [2, 3].

Clinical studies at the beginning of the twenty-first century showed that patients with advanced NSCLC benefit from platinum-based doublet therapy including third-generation drugs (vinorelbine, gemcitabine, taxanes) with improved survival and quality of life as compared to palliative support therapy [4]. Therefore, the ESMO (European Society for Medical Oncology) consensus conference on lung cancer and ESMO clinical practice guidelines recommend chemotherapy with platinum doublets as treatment for all stage IV NSCLC patients with epidermal growth factor receptor (EGFR)- and anaplastic lymphoma kinase (ALK)-negative disease [3, 5].

Bevacizumab (Avastin®), a recombinant humanized monoclonal antibody, binds selectively to the human vascular endothelial growth factor (VEGF) and thereby inhibits the binding of VEGF to its receptors, Flt-1 (FMS-related tyrosine kinase 1, VEGF receptor [VEGFR]-1) and KDR (kinase insert domain receptor, VEGFR-2) on the surface of endothelial cells. Neutralizing the biological activity of VEGF causes regression of the vascularization of tumors, normalizes remaining tumor vasculature, and inhibits the formation of new tumor vasculature, thereby inhibiting tumor growth [6]. Safety and efficacy of bevacizumab in combination with a platinum-based chemotherapy in first-line (1 L) treatment of patients with non-squamous NSCLC were investigated in the phase III trials E4599 and BO17704 (AVAiL) [7, 8].

In the randomized, open-label E4599 study, patients received chemotherapy alone (carboplatin and paclitaxel; “CP” arm) or in combination with bevacizumab at a dose of 15 mg/kg body weight (“CP + bevacizumab” arm). After completion of six cycles or upon premature discontinuation of chemotherapy, patients on the “CP + bevacizumab” arm continued to receive bevacizumab as a single agent every 3 weeks until disease progression. Overall survival (OS) was improved in the “CP + bevacizumab” group in comparison to the “CP” group. Median progression-free survival (PFS) was also increased, with corresponding response rates [7]. Based on the results of the E4599 study, FDA (Food and Drug Administration) approval was obtained for bevacizumab in combination with CP for 1 L treatment of adult patients with non-resectable advanced, metastatic or recurrent NSCLC in 2006 [7, 9]. In September 2007 marketing authorization in this indication was granted for the European Union [10].

The randomized, placebo-controlled, double-blind BO17704 study (AVAiL) evaluated cisplatin and gemcitabine (CG) plus bevacizumab vs. CG plus placebo in patients with advanced, non-squamous NSCLC who had not received prior chemotherapy. After up to six cycles with CG (cisplatin: 80 mg/m^2^, gemcitabine: 1250 mg/m^2^) plus bevacizumab (at a dose of 7.5 mg/kg or 15 mg/kg) or plus placebo, patients of the bevacizumab-including arms were allowed to receive bevacizumab as single-agent until disease progression or unacceptable toxicity. BO17704 demonstrated that both bevacizumab doses increased PFS and objective response rates as compared to placebo [8].

Systematic reviews and meta-analyses [4, 11, 12] showed a consistent significant improvement of response rates, PFS, and OS for the combination of bevacizumab and platinum-based chemotherapy compared with platinum-based chemotherapy alone in patients with non-squamous NSCLC. Therefore, guidelines recommend that the incorporation of bevacizumab into individual treatment schedules along with platinum-based chemotherapies should be considered in eligible patients [5, 13], and the addition of bevacizumab to systemic chemotherapy has become a standard of care for the 1 L treatment of patients in many institutions [4, 14, 15].

In clinical trials for NSCLC, the following side effects occurred more often in patients receiving bevacizumab plus chemotherapy than chemotherapy alone: bleeding (epistaxis, hemoptysis), hypertension, proteinuria, and neutropenia. Additional common side effects include fatigue, asthenia, diarrhea, and abdominal pain [16].

After approval of bevacizumab for treatment of NSCLC in combination with platinum doublets, the use of bevacizumab expanded into daily routine, no longer adhering to specific inclusion and exclusion criteria defined for clinical trials. Phase IV single-arm and observational studies were conducted to obtain information on real-world safety, effectiveness, and usage patterns of bevacizumab. Although the results from such studies are not directly comparable with randomized controlled trials (RCTs), they add useful information on clinical routine practice to the existing body of knowledge [15].

The ARIES observational cohort study included a real-world patient population with 1 L bevacizumab treatment plus chemotherapy. The incidences of bevacizumab–associated adverse events were consistent with those in RCTs and so were the results regarding PFS and OS [15]. The open-label, single-arm, multicenter phase IV study SAiL of 1 L treatment of advanced NSCLC demonstrated a median time to disease progression (TTP) and median OS exceeding the results of the previous trial observations without identifying new safety signals [17].

The present post-authorization non-interventional study (NIS) AVAiLABLE – bevacizumab (Avastin®) in lung cancer was part of the marketing authorization holder’s post-approval commitment for further pharmacovigilance surveillance. The prospective cohort study had the objective to evaluate the effectiveness and safety of intravenous (i.v.) bevacizumab in combination with platinum-based chemotherapy as 1 L treatment of patients with non-resectable, advanced, metastatic or recurrent, predominantly non-squamous NSCLC under routine conditions in Germany. Special attention was given to patients with/without adenocarcinoma and the potential benefit in elderly patients.

## Methods

### Patients and study design

The post-authorization NIS AVAiLABLE (ClinicalTrials.gov Identifier: NCT02596958) was planned to include 900 patients with non-resectable, advanced, metastatic or recurrent NSCLC other than predominantly squamous cell lung cancer across Germany. Further selection criteria included: (1) age ≥ 18 years, (2) histologically confirmed non-squamous NSCLC, (3) no contraindication to bevacizumab according to current label, (4) therapeutic decision for 1 L treatment with bevacizumab combined with platinum-based chemotherapy made independently from this NIS. The planned observation period was 51 weeks. No study-specific treatments or assessments were scheduled. A study observation interval of maximally six cycles of chemotherapy plus bevacizumab, followed by bevacizumab maintenance therapy until disease progression was recommended. However, according to the non-interventional approach, actual treatment decisions were at the discretion of the treating physicians. Normal merchandise was used and reimbursed by the respective national or private health insurance. The observational plan was evaluated by the Ethics Committee of the Medical Association of Lower Saxony (‘Ärztekammer Niedersachsen’) in Hannover (Germany) and the study performed in accordance with the Declaration of Helsinki and Good Clinical Practice guidelines.

The study was conducted across 185 institutions in Germany (medical oncologists and pneumologists in hospitals and private practices) between September 2007 and October 2013. Patients were followed until progression or intolerable toxicity, whichever occurred first. The final documentation was to be performed within 4 weeks after end of bevacizumab treatment, regardless of further therapy options. No long-term follow-up information on deaths was collected. Quality control procedures were applied to each stage of data entry and data handling to ensure that all data were reliable and processed correctly. A data review meeting was held prior to database lock.

Effectiveness endpoints were response rates, PFS, and OS in a large, unselected patient population. Safety endpoints comprised occurrence and frequency of adverse drug reactions (ADRs; also ADRs of special interest; see next section) including seriousness, relatedness to bevacizumab and severity. Additional safety endpoints were the occurrence of any new or rare bevacizumab-related ADRs as well as ADRs leading to treatment discontinuation. Pre-specified subgroup analyses of effectiveness and safety endpoints were performed for patients with/without adenocarcinoma and various age groups. Differences between groups were not tested for statistical significance. Further research questions included patient characteristics at baseline (e.g. demographic data, cancer history) and details on treatment with bevacizumab and chemotherapy (dose, regimen, duration).

### Variables and data sources

Baseline information collected prospectively per patient included demographics and cancer history, current tumor status, previous treatment, and relevant concomitant diseases. The initial diagnosis including adenocarcinoma had to be confirmed histopathologically. Vital signs, standard laboratory assessments, and general condition were also captured at baseline.

During treatment (every 3 weeks), details on the systemic therapy with bevacizumab (daily dose, infusion time, dose deviations and therapy interruptions) and concomitant antineoplastic agents were recorded. Tumor staging and Eastern Cooperative Oncology Group performance status (ECOG PS) were assessed as per clinical routine at the individual center. ADRs and toxicity based on NCI/CTC (National Cancer Institute/Common Terminology Criteria) were reported at each visit retrospectively for the period since the previous visit. Data regarding adverse events, previous and concomitant diseases were coded using the Medical Dictionary for Regulatory Activities (MedDRA) version 15.1; medications were coded using the World Health Organization drug dictionary (WHO DD) version 4.1.

ADRs of special interest included hypertension, hemorrhages, gastrointestinal perforation/fistula, tracheoesophageal fistula, proteinuria, wound healing complications, congestive heart failure, hemoptysis, and thromboembolism. Serious ADRs and ADRs of special interest had to be reported on separate report forms within 24 h of notice. Best tumor response over time, reasons for end of therapy, and further antineoplastic therapy were documented at an end of study visit.

### Statistics

A sample size of 900 patients was estimated to allow a 99% probability to record an ADR with a true incidence of 1% at least twice. Data were evaluated using descriptive statistical methods. Missing values were not replaced. Specification of the complete analysis was laid down in detail in the statistical analysis plan, which was finalized prior to database lock. Time-to-event analyses were performed using Kaplan Meier methodology and include the corresponding 95% confidence intervals (CIs).

PFS was defined as time from start of therapy to investigator-assessed disease progression or death from any cause. OS was defined as time from treatment start to death if death occurred within the time window between start of therapy and up to four weeks after last bevacizumab administration (final documentation). Patients without event (progression or death) were censored at the end of study or data cut-off date (whichever occurred first). The disease control rate (DCR), defined as percentage of patients achieving complete response (CR), partial response (PR), and/or stable disease (SD) during the course of the observation, was calculated as further effectiveness endpoint.

For patients with NSCLC other than non-squamous (documented as yes vs. no), subgroup analyses evaluated the effect on PFS by age (< 65 vs. 65 to < 70 vs. 70 to < 75 vs. ≥75 years), sex (male vs. female), presence of adenocarcinoma (yes vs. no/unknown), dose level (7.5 mg/kg vs. 15 mg/kg vs. other doses), TNM-stage (stage III vs. stage IV vs. residual), presence of distant metastases, prior treatment (with vs. without operation, radiation, chemotherapy, other), general condition (ECOG PS), best overall response rate (ORR; SD vs. PR vs. CR), number of cycles with bevacizumab, and number of cycles with maintenance therapy. Incidences and the number of episodes were calculated for ADRs and subgroup analyses were performed for grade ≥ 3 toxicity events by age group (< 65 vs. 65 to < 70 vs. 70 to < 75 vs. ≥75 years), and adenocarcinoma (yes vs. no/unknown).

## Results

### Patient characteristics at baseline

A total of 996 patients consented to the collection and processing of their personal data, nine patients were excluded due to second-line (2 L) treatment. The analysis population included 987 NSCLC patients who were evaluated in the NIS AVAiLABLE. Mean (± standard deviation, σ) patient age was 61.5 (±9.8) years. 58.3% of the patient population was below 65 years. Male patients accounted for 59.8% of the study population. The average body weight of all patients was 74.8 (±18.6) kg, mean body mass index (BMI) was 25.1 (±4.5) kg/m^2^, and 88.3% of patients had ECOG PS 0 or 1 (i.e. no or only minor restrictions in physical activity). 72.8% of the patients were diagnosed with stage IV disease at the start of observation. A proportion of patients had bone metastases (35.3%), malignant pleural effusion (26.9%), liver metastases (18.3%), adrenal gland metastases (14.7%), and/or brain metastases (10.2%). The majority of patients (90.1%; *N* = 953 non-missing data) had histologically confirmed adenocarcinoma. Recorded separately, 80.8% (*N* = 874 non-missing data) met the bevacizumab label ‘NSCLC other than predominantly squamous cell histology’. Baseline characteristics regarding age, gender, ECOG PS, adenocarcinoma/non-squamous cell histology, and tumor stage are displayed in Table 1.

**Table 1:** Patient Characteristics (Analysis Population)

Patient Characteristics	*n* (%)
Mean age ± σ [years]	61.5 ± 9.8
Age group, *N* = 972	
< 65 years	567 (58.3)
65 to < 70 years	199 (20.5)
70 to < 75 years	134 (13.8)
≥ 75 years	72 (7.4)
Gender, *N* = 986	
Female	396 (40.2)
Male	590 (59.8)
ECOG PS, *N* = 894	
0	315 (35.2)
1	474 (53.0)
2	99 (11.1)
3	6 (0.7)
Adenocarcinoma histology, *N* = 953	
Yes	859 (90.1)
No	74 (7.8)
Unknown	20 (2.1)
NSCLC other than predominantly squamous cell histology, *N* = 874	
Yes	706 (80.8)
No	168 (19.2)
Tumor stage at start of observation, *N* = 903	
Inoperable	246 (27.2)
Stage IV	657 (72.8)
TNM-staging at initial diagnosis, *N* = 864	
IA	14 (1.6)
IB	22 (2.5)
IIA	6 (0.7)
IIB	17 (2.0)
IIIA	60 (6.9)
IIIB	43 (5.0)
IV	702 (81.3)

Percentages refer to all patients with non-missing data, TNM: Tumor-Node-Metastasis

### Treatment

Between study start on 28 September 2007 and study end on 4 October 2013, patients were observed for a mean time (±σ) of 197.6 (±177.4) days. Mean duration (±σ) of bevacizumab therapy was 7.6 (±7.0) cycles; on average patients received 4.2 (±1.8) cycles of combination therapy and 3.4 (±6.2) cycles of bevacizumab maintenance therapy. Overall 45.8% of patients received single-agent bevacizumab maintenance therapy. For all treatment cycles (combination and bevacizumab maintenance therapy) the number of patients per cycle decreased from 978 (99.1%) at the beginning to 117 (11.9%) at treatment weeks 49 to 51 (reasons for the end of therapy are described in the *Safety* section). Bevacizumab doses per infusion were mostly 5, 7.5, 10, or 15 mg/kg body weight; the median dose throughout all cycles was 7.5 mg/kg. More than 65% of patients were on the 7.5 mg dose regimen; about 20% of the patients received the 15 mg dose up to treatment week 18.

The main therapy combination used was bevacizumab plus carboplatin and paclitaxel (38.0%). In a smaller proportion of patients, bevacizumab was combined with carboplatin and pemetrexed (11.7%) or cisplatin and pemetrexed (10.1%). Besides these three most commonly used regimens, other platinum combinations were used as well. All these regimens are following current recommendations for doublet therapy with platinum including third-generation drugs [3]. Only 9.3% of patients received chemotherapy regimens not containing platinum compounds.

### Clinical effectiveness

Within the analysis population, the majority of patients achieved PR (43.3%) or SD (29.4%) before progression or intolerable toxicity; CR was reached by 2.3%. The DRC (percentage of patients who achieved CR, PR or SD during observation) was 75.0% and the ORR (CR plus PR) was 45.6% (Table 2).

**Table 2:** Response Rates (Analysis Population)

Response	*N* = 976
ORR [%]	45.6
CR, complete response: *n* (%)	22 (2.3)
PR, partial response: *n* (%)	423 (43.3)
SD, stable disease: *n* (%)	287 (29.4)
PD, progressive disease: *n* (%)	99 (10.1)
Not evaluable: *n* (%)	145 (14.9)
DCR [%]	75.0

**Fig. 1:**
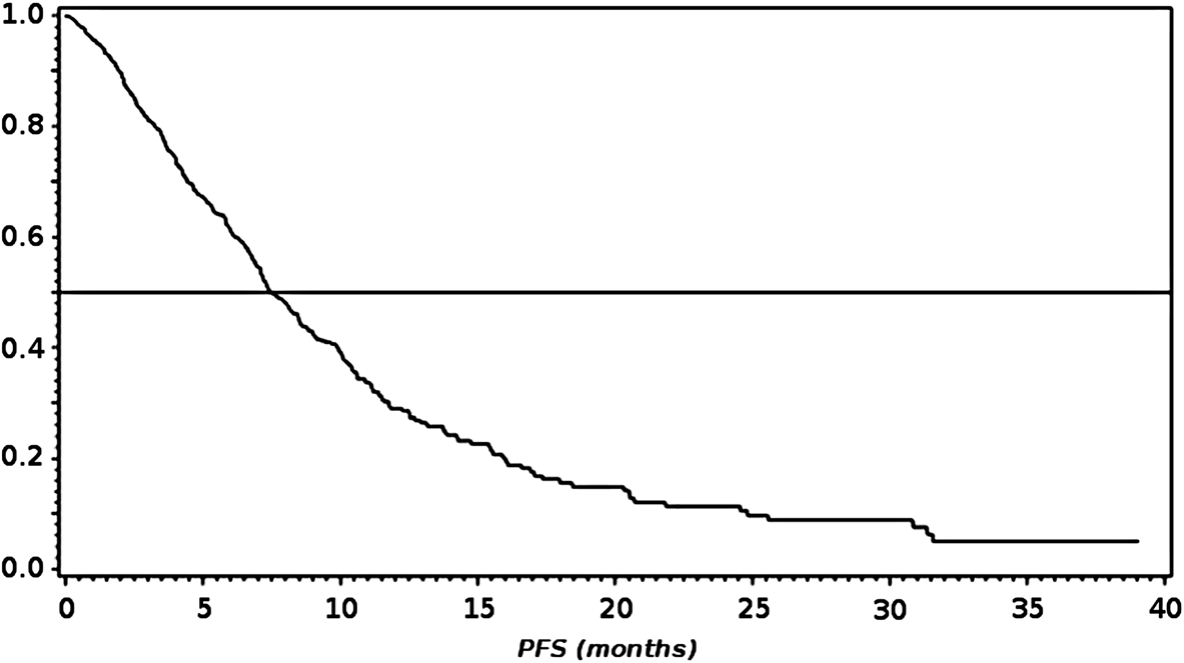
Kaplan Meier Analysis of Progression-free Survival (PFS, Analysis Population)

Kaplan Meier estimate of time-to-progression resulted in a median PFS of 7.4 months (95% CI: 7.1; 8.4) for the analysis population. 50% of patients were within the range of 3.9 and 13.8 months until estimated disease progression (Fig. 1). In the subgroup of TMN stage IV patients with NSCLC other than predominantly squamous cell histology (*N* = 492), median PFS was 7.1 months (95% CI: 6.7; 8.0).

The only follow-up was performed four weeks after the end of treatment with bevacizumab. At that time, the mean OS was 18.4 months (±0.5). However, 82.5% of the analysis population were alive four weeks after the end of bevacizumab treatment and had to be censored. Therefore, the OS estimate is based on a low number of events and does not allow for a meaningful interpretation.

Subgroup analyses performed for 706 patients with NSCLC other than predominantly squamous cell histology revealed that longer PFS was associated with the presence of adenocarcinoma at baseline in comparison with other histological NSCLC subgroups (Table 3). For 314 patients receiving maintenance therapy with bevacizumab, the median PFS was 10.9 months (95% CI: 10.0; 12.5) with 50% of the patients within the range of 7.1 and 17.4 months.

**Table 3:** Subgroup Analysis of Median PFS by Age, Presence or Absence of Adenocarcinoma, and Chemotherapy Combination

	*n*	Median PFS (95% CI)
Age [years]		
< 65	380	8.7 (7.4;10.1)
65 to < 70	150	6.8 (5.8; 9.9)
70 to < 75	96	8.0 (6.1; 8.6)
≥ 75	47	7.4 (5.3; 20.5)
Histologically confirmed adenocarcinoma		
Yes	596	8.2 (7.2; 9.7)
No / unknown	61	5.0 (4.3; 9.1)
Chemotherapy combined with bevacizumab		
Carboplatin/gemcitabine	76	7.7 (5.8; 10.1)
Carboplatin/pemetrexed	108	8.2 (6.0; 12.5)
Carboplatin/paclitaxel	352	7.4 (6.7; 8.8)
Cisplatin/gemcitabine	87	7.0 (5.1; 10.0)
Cisplatin/pemetrexed	95	7.2 (4.6; 10.1)
Cisplatin/vinorelbine	59	7.3 (5.3; 14.2)
Other carboplatin combinations^a^	128	9.0 (7.1; 11.4)
Other cisplatin combinations^b^	64	9.1 (7.4; 14.2)
Combinations not containing platinum	87	8.5 (5.4; 11.5)

For age and adenocarcinoma the number of patients refers to patients with non-squamous NSCLC (*N* = 706) and available data; for chemotherapy the data refer to the analysis population (N = 987) with available data. For chemotherapy combinations only subgroups with *n* > 50 patients are displayed; multiple counts were possible

^a^Other carboplatin combinations include bevacizumab/carboplatin, bevacizumab/carboplatin/docetaxel, bevacizumab/carboplatin/vinorelbine, and carboplatin/paclitaxel without bevacizumab (only subgroups with n ≥ 10 patients mentioned)

^b^Other cisplatin combinations include bevacizumab/cisplatin and bevacizumab/cisplatin/paclitaxel (only subgroups with *n* ≥ 10 patients mentioned)

### Safety

A total of 4991 ADRs were observed in 874/987 patients (88.6%). 110/987 patients (11.1%) experienced serious ADRs that led to premature study termination in 44/987 patients (4.6%). In 266/987 patients (27.0%), an ADR was considered treatment-related to bevacizumab.

Overall, ADRs with grade ≥ 3 were reported for 332 patients (33.6%). Table 4 gives an overview of grade ≥ 3 ADRs by Preferred Term occurring in ≥1% of the analysis population and for the subgroups by age. In the age group 70 to < 75 years, a tendency of increased occurrence of grade ≥ 3 ADRs was observed for hematological parameters (anemia, leukopenia, neutropenia, thrombocytopenia), nausea, pain, and pain in extremity; only hypertension grade ≥ 3 occurred more often in the age group ≥75 years than in age classes with patients < 65 years and < 75 years (Table 4). No substantial differences were observed in grade ≥ 3 ADRs between patients with confirmed adenocarcinoma and patients with other histological findings (data not shown).

**Table 4:** ADRs Grade ≥ 3 Occurring in ≥1% of the Analysis Population by Age Subgroups

Grade ≥ 3 ADRs	*n* (%)				
Age groups [years]	< 65 (*N* = 567)	65 to < 70 (*N* = 199)	70 to < 75 (*N* = 134)	≥ 75 (*N* = 72)	Analysis Population (*N* = 987)
Any	196 (34.6%)	65 (32.7%)	51 (38.1%)	18 (25.0%)	332 (33.6%)
Leukopenia	64 (11.3%)	25 (12.6%)	25 (18.7%)	6 (8.3%)	120 (12.2%)
Neutropenia	67 (11.8%)	22 (11.1%)	18 (13.4%)	7 (9.7%)	115 (11.7%)
Thrombocytopenia	47 (8.3%)	14 (7.0%)	13 (9.7%)	3 (4.2%)	78 (7.9%)
Anemia	34 (6.0%)	4 (2.0%)	9 (6.7%)	2 (2.8%)	50 (5.7%)
Nausea	20 (3.5%)	1 (0.5%)	7 (5.2%)	3 (4.2%)	31 (3.1%)
Vomiting	11 (1.9%)	2 (1.0%)	1 (0.7%)	2 (2.8%)	16 (1.6%)
Diarrhea	8 (1.4%)	1 (0.5%)	0 (0.0%)	2 (2.8%)	11 (1.1%)
Pain	11 (1.9%)	2 (1.0%)	4 (3.0%)	1 (1.4%)	18 (1.8%)
Chest pain	3 (0.5%)	4 (2.0%)	3 (2.2%)	0 (0.0%)	10 (1.0%)
Pain in extremity	6 (1.1%)	3 (1.5%)	4 (3.0%)	1 (1.4%)	14 (1.4%)
Back pain	6 (1.1%)	3 (1.5%)	2 (1.5%)	1 (1.4%)	12 (1.2%)
Peripheral sensory neuropathy	9 (1.6%)	3 (1.5%)	1 (0.7%)	1 (1.4%)	14 (1.4%)
Hypertension	8 (1.4%)	4 (2.0%)	1 (0.7%)	3 (4.2%)	16 (1.6%)

Bevacizumab-related ADRs grade ≥ 3 occurred in 56 patients (5.7% of the analysis population) with the highest incidence for leukopenia (11 patients, 1.1%), neutropenia (9 patients, 0.9%), and hypertension (10 patients, 1.0%). Patients in the age group ≥75 years and patients with adenocarcinoma were slightly more affected by bevacizumab-related ADRs (data not shown).

Incidences of bevacizumab-related ADRs of special interest were: hypertension 7.0% (grade ≥ 3: 1.0%), proteinuria 3.6% (grade ≥ 3: 0.1%), hemorrhage 0.1% (none grade ≥ 3), hemoptysis 1.4% (grade ≥ 3: 0.2%), gastrointestinal perforation 0.1% (all grade ≥ 3), large intestine perforation 0.3% (grade ≥ 3: 0.1%) and esophagobronchial fistula 0.1% (all grade ≥ 3).

The main reason for the end of therapy was disease progression in 443 (46.4%) patients. 138 (14.5%) patients died from the underlying disease and 37 (3.9%) patients from another cause (without further specification).

Maintenance therapy with bevacizumab was well tolerated and safe, even for more than 20 cycles (median PFS 31.6 months in 12 patients). No new safety signals were observed in this NIS.

## Discussion

### Comparison with other studies

To place the present results within the context of previous research, Table 5 gives an overview of the key results of studies investigating bevacizumab 1 L chemotherapy for NSCLC. It compares the NIS AVAiLABLE with the NIS ARIES [15, 18], the phase IV study SAiL [17, 19], and the RCTs AVAiL [8, 20] and E4599 [7, 21].

**Table 5:** Comparison of Key Parameters in Trials with Bevacizumab 1 L Chemotherapy for NSCLC

	AVAiLABLE (*N* = 987)	ARIES (*N* = 1967)	SAiL (*N* = 2212)	AVAiL (*N* = 345) / (*N* = 351)	E4599 (*N* = 434)
Study design	NIS	NIS	Phase IV	RCT	RCT
Study duration	09/2007–10/2013	11/2006–03/2012	08/2006–07/2009	02/2005–10/2006	07/2001–04/2004
Bevacizumab dosage	Investigator’s choice: 65% pts. 7.5 mg/kg, 20% pts. 15 mg/kg, 15% pts. other dosage; median dose: 7.5 mg/kg	Investigator’s choice	Investigator’s choice: 261 (12%) pts. 7.5 mg/kg, 1947 (88%) pts15 mg/kg	7.5 mg/kg / 15 mg/kg	15 mg/kg
Chemotherapy regimen, patients (%)	Investigator’s choice: 90.7% platinum regimens	Investigator’s choice: 89.9% (1768 pts) platinum-doublets	Investigator’s choice: 86% (1916 pts) platinum-doublets	per protocol: cisplatin + gemcitabine	per protocol: carboplatin + paclitaxel
Adenocarcinoma histology, patients (%)	859 (90.1%)	1363 (69.3%)	1781 (86%)	293 (85%) / 300 (85%)	366 (88%)
ORR (%)	45.6 (based on 976 pts)	49.0	51.5	37.8 /34.6	35
Overall median PFS/TTP, [months] (95% CI)	PFS 7.4 (7.1–8.4) (based on 423 pts)	PFS 6.6 (6.3–6.9)	TTP 7.8 (7.5–8.1)	PFS 6.7 / PFS 6.5 [8]	PFS 6.2
Bevacizumab maintenance	314 pts. (45%, based on 697 pts), thereof 166 (53%) more than 6 cycles	530 pts. post induction therapy [18]	1047 pts. post induction therapy [19]	145 pts. (42%) / 145 pts. (41%)	215 pts. (53%), thereof 107 (50%) more than 5 cycles
Median OS, [months] (95% CI)	not applicable	13.0 (12.2–13.8)	14.6 (13.8–15.3)	13.6 (11.8–15.8)/13.4 (11.1–15.1) [20]	12.3
Median treatment duration with bevacizumab, (range)	125.5 (approx. 17.9 weeks) (1–1332) days; 6.0 cycles (0–86 cycles)	3.7 (0.1–49.3) months (approx. 15.9 weeks); 6 doses (1–88 doses)	21.3 weeks (0.1–132.3; 9.1–36.4)	4.9 cycles (approx. 14.7 weeks)/4.4 cycles (approx. 13.2 weeks)	7 cycles (approx. 21 weeks)
Duration of follow-up (FU)	one month FU after last bevacizumab administration per patient	median 12.5 months (range 0.2–65.5)	mean 380.4 days (±215.8 days; 12.5 months)	12.5 months/12.9 months	2-years OS Data [21]
Safety	bevacizumab-related ADRs: 266 (27.0%); total ADRs: 4991; pts. with ADRs: 874 (88.6%)	grade ≥ 3 bevacizumab-related AE incidence: 19.7%	AEs of special interest for bevacizumab: 3821	grade ≥ 3 events: 252 (76%)/265 (81%)	only events grade ≥ 3 and hematologic events grade ≥ 4: 263

Pts: Patients, Approx: Approximately, AEs: Adverse events

With regard to the effectiveness outcomes ORR and PFS, the AVAiLABLE results are consistent with previous studies. Of note, no new safety signals were observed in this NIS. The number of ADRs observed during the AVAiLABLE NIS cannot be compared directly with the safety data reported from controlled clinical trials due to varying documentation requirements in different study types. During controlled clinical trials, usually all adverse events are reported regardless of causal relationship to study treatment.

### Potential Bias and limitations

Results from clinical trials are prone to selection bias due to specific inclusion and exclusion criteria. Moreover, their pre-specified diagnostic and follow-up measures are not always representative approaches of day-to-day practice [22]. In comparison, a NIS is an appropriate method of gathering real-world data for effectiveness and tolerability of a listed drug under routine conditions, although a potential bias related to data being incorrectly reported, transcribed, or missing and a remaining selection bias cannot be ruled out.

In the study at hand, only 706 out of 859 patients included with adenocarcinoma at baseline actually met the bevacizumab label ‘NSCLC other than predominantly squamous cell histology’ (Table 1).

A single follow-up visit was planned at four weeks after the last bevacizumab administration; no further documentation of survival data was scheduled as per protocol. 804 (82.5%) patients were still alive at the time point of last documentation and had to be censored for OS analysis. Due to the short follow-up period, this NIS does not provide reasonable conclusions regarding OS.

### Study results in the context of current treatment recommendations

In the context of other major studies investigating bevacizumab in combination with 1 L chemotherapy for patients with advanced non-squamous NSCLC, the NIS AVAiLABLE confirms the effectiveness and safety of bevacizumab therapy. Although the study started in 2007, the platinum-doublet chemotherapy used for 1 L treatment in advanced NSCLC is still the current first choice and has become routine clinical practice. According to recent guidelines, combination with bevacizumab and other platinum-based chemotherapies may be considered in eligible patients (non-small-cell cancer and ECOG PS 0–1) and bevacizumab may be added to carboplatin plus paclitaxel if no contraindications exist [5, 13]. Subgroup analyses for patients with NSCLC other than predominantly squamous cell histology show effectiveness for bevacizumab in all chemotherapy combinations containing platinum. As this NIS was conducted under real-life conditions with choice of the chemotherapy regimen at the investigator’s discretion, the number of 90 (9.7%) patients not receiving the recommended platinum treatment may not come as a surprise although specified otherwise in the inclusion criteria. It is in accordance with the results of the observational ARIES and phase IV SAiL studies [15, 17] and may be attributed to patients unsuitable for platinum therapy due to age, concomitant diseases, or performance status.

Pre-specified subgroup analyses focused on patients with/without adenocarcinoma and different age groups (Table 3). Median PFS was 3 months longer in patients with adenocarcinoma compared to other histological NSCLC subgroups. All age groups benefit from bevacizumab treatment. Overall, maintenance therapy with bevacizumab was well tolerated and safe, even for more than 20 cycles (median PFS 31.6 months in 12 patients).

### Other cancer immunotherapy options

Other targeted therapies with VEGFR antibodies (aflibercept, ramucirumab) and tyrosine kinase inhibitors with selectivity for VEGFRs (sorafenib, sunitinib, nintedanib, cediranib, motesanib, pazopanib, axitinib, vandetanib) showed responses and improved PFS but no survival advantages in patients with advanced NSCLC [4]. Therefore, the established standard of care of bevacizumab in combination with carboplatin/paclitaxel in the 1 L setting, and ramucirumab in combination with docetaxel in the 2 L setting are recommended by recent guidelines. In addition, substantial progress resulted from cancer immunotherapies targeting either the programmed death ligand 1 (PD-L1) or the programmed death-1 (PD-1) pathway in patients with NSCLC. Based on new data from clinical trials in NSCLC, immunotherapies form a new standard in 2 L treatment (nivolumab, pembrolizumab) [23, 24], or 1 L treatment (pembrolizumab) [25] of patients expressing PD-L1, and possibly even for those who do not.

The combination of cancer immunotherapies, including bevacizumab, may amplify the immune system’s ability to eliminate cancer [26]. Current and future roles of bevacizumab in the 1 L therapy of NSCLC include combination therapy with erlotinib and bevacizumab in populations with EGFR-mutations [27, 28] as well as combination therapy with atezolizumab and bevacizumab [29].

## Conclusions

In conclusion, the observed real-world effectiveness and safety data of the NIS AVAiLABLE investigating bevacizumab in combination with a broad range of 1 L treatment regimens support and complement existing bevacizumab data. As single-agent maintenance therapy, bevacizumab was shown to be well tolerated and safe even for long treatment periods. All in all, bevacizumab is generally recognized as an approved element of advanced NSCLC treatment, recommended by current guidelines, and with potential to show consistent efficacy in treatment combinations.

## Abbreviations

1 L: first-line
2 L: second-line
ADR: Adverse Drug Reaction
ALK: Anaplastic Lymphoma Kinase
BMI: Body Mass Index
CG: Cisplatin and Gemcitabine
CI: Confidence Interval
CP: Carboplatin and Paclitaxel
CR: Complete Response
DCR: Disease Control Rate
ECOG PS: Eastern Cooperative Oncology Group Performance Status
EGFR: Epidermal Growth Factor Receptor
ESMO: European Society for Medical Oncology
FDA: Food and Drug Administration
Flt-1: FMS-Related Tyrosine Kinase 1
i.v: intravenous
KDR: Kinase Insert Domain Receptor
MedDRA: Medical Dictionary for Regulatory Activities
NCI/CTC: National Cancer Institute/Common Terminology Criteria
NIS: Non-Interventional Study
NSCLC: Non-Small-Cell Lung Cancer
OS: Overall Survival
ORR: Overall Response Rate
PFS: Progression-Free Survival
PR: Partial Response
RTC: Randomized Controlled Trial
σ: Standard Deviation
SD: Stable Disease
TTP: Time to Disease Progression
VEGF: Vascular Endothelial Growth Factor
VEGFR-1: Vascular Endothelial Growth Factor Receptor-1
VEGFR-2: Vascular Endothelial Growth Factor Receptor-2
WHO DD: World Health Organization drug dictionary
